# 5G-enabled smart hospitals: Innovations in patient care and facility management

**DOI:** 10.1097/MD.0000000000038239

**Published:** 2024-05-17

**Authors:** Chukwuka Elendu, Tochi C. Elendu, Ijeoma D. Elendu

**Affiliations:** a Federal University Teaching Hospital, Owerri, Nigeria; b Imo State University, Owerri, Nigeria.

**Keywords:** 5G technology, facility management, healthcare innovation, patient care, smart hospitals

## Abstract

Smart hospitals represent the pinnacle of healthcare innovation, leveraging cutting-edge technologies to transform patient care and facility management. This article addresses the pressing need for effective implementation of 5G technology in smart hospitals, aiming to enhance connectivity, improve patient outcomes, and drive operational efficiency. The methodology employed involves a comprehensive review of existing literature, case studies, and expert insights to analyze the impact of 5G on various aspects of smart hospital operations. The article highlights the significance of 5G technology in enabling real-time data analytics, remote monitoring, and telemedicine, thus revolutionizing healthcare delivery. By providing high-speed, low-latency connectivity, 5G facilitates seamless communication and collaboration among healthcare providers, leading to more efficient diagnosis, treatment, and patient care. Additionally, the adoption of 5G enables smart hospitals to leverage artificial intelligence (AI)-based solutions for predictive analytics, personalized medicine and enhanced patient engagement. Furthermore, the article explores the potential of 5G-enabled smart hospitals in enhancing disaster preparedness and emergency response efforts. Case studies and examples demonstrate how 5G technology can improve situational awareness, coordinate resources, and deliver timely care during natural disasters and pandemics. Overall, this article underscores the transformative impact of 5G technology on smart hospitals and emphasizes the importance of embracing innovation to meet the evolving needs of patients and communities. By adopting 5G technology, smart hospitals can usher in a new era of healthcare delivery characterized by enhanced connectivity, improved patient outcomes, and unparalleled efficiency.

## 1. Introduction

Smart hospitals, at the forefront of healthcare innovation, integrate advanced technologies to revolutionize patient care and facility management.^[1]^ This article addresses the need to implement 5G technology effectively in smart hospitals to enhance connectivity, improve patient outcomes, and drive operational efficiency.^[2]^ With the proliferation of internet of things (IoT) devices, artificial intelligence (AI), and real-time data analytics, smart hospitals are poised to deliver unparalleled healthcare experiences. However, the full potential of these innovations can only be realized with the seamless integration of high-speed, low-latency connectivity provided by 5G networks.^[3]^

The primary problem addressed in this article lies in the gap between the potential of 5G technology and its practical implementation in smart hospitals. Despite the promising capabilities of 5G, challenges such as infrastructure readiness, interoperability issues, and regulatory constraints hinder its widespread adoption in healthcare settings.^[4]^ By identifying and addressing these barriers, healthcare organizations can unlock the transformative power of 5G to enhance patient care delivery and facility management.

The contributions of our work lie in the comprehensive analysis of existing literature, case studies, and expert insights to elucidate the impact of 5G on smart hospital operations.^[5]^ Through a systematic review of relevant research articles and industry reports, we provide a nuanced understanding of the opportunities and challenges associated with 5G implementation in healthcare. Furthermore, our article offers practical recommendations and strategies for healthcare stakeholders to navigate the complexities of 5G adoption and leverage its potential to drive innovation and excellence in patient care.^[1]^

The novelty of our work stems from its focus on the intersection of 5G technology and smart hospitals, a rapidly evolving field with significant implications for the future of healthcare delivery.^[1]^ By synthesizing the latest advancements in 5G technology with real-world examples and case studies, we provide valuable insights into the transformative potential of 5G-enabled smart hospitals.^[6]^ Through our rigorous analysis and forward-thinking approach, we aim to inspire healthcare leaders, policymakers, and technology innovators to embrace 5G as a catalyst for positive change in healthcare.

## 2. Discussion

### 2.1. Overview of smart hospitals

In the rapidly evolving healthcare landscape, smart hospitals stand at the forefront of innovation, leveraging cutting-edge technologies to transform traditional healthcare delivery models into intelligent and interconnected ecosystems.^[5]^ At its essence, a smart hospital integrates many advanced technologies, including IoT devices, AI, cloud computing, and data analytics, to optimize patient care, enhance operational efficiency, and improve overall healthcare outcomes.^[1]^

The integration of IoT devices plays a pivotal role in the functionality of smart hospitals, enabling the seamless connection and communication of medical devices, sensors, and equipment throughout the healthcare facility.^[2]^ These IoT devices can range from wearable monitors and implantable sensors to smart beds and medication dispensers, each collecting real-time data on patient vital signs, medication adherence, and environmental conditions.^[5]^ By aggregating and analyzing this data, healthcare providers can gain valuable insights into patient health status, identify potential risks or abnormalities, and deliver more personalized and proactive care.

In addition to IoT devices, AI technologies are increasingly deployed in healthcare settings to augment clinical decision-making, automate routine tasks, and improve diagnostic accuracy. Machine-learning algorithms, for example, can analyze vast amounts of medical imaging data to assist radiologists in detecting anomalies or predicting disease progression.^[2]^ Natural language processing algorithms can extract relevant information from electronic health records to support clinical documentation and streamline administrative workflows. Moreover, AI-powered virtual assistants and chatbots can enhance patient engagement, provide personalized health recommendations, and offer round-the-clock support for routine inquiries.^[6]^

Central to the functioning of smart hospitals is the importance of connectivity, particularly high-speed and reliable network infrastructure, such as 5G technology. The adoption of 5G enables seamless communication and data exchange between various devices and systems within the healthcare facility, facilitating real-time monitoring, remote consultations, and telemedicine services.^[7]^ With ultra-low latency and high-bandwidth capabilities, 5G networks empower healthcare providers to deliver timely interventions, access critical patient information, and collaborate with multidisciplinary teams regardless of geographical constraints.

In essence, smart hospitals represent the convergence of advanced technologies and healthcare expertise aimed at redefining the patient experience, optimizing clinical workflows, and driving continuous innovation in healthcare delivery. By harnessing the power of IoT, AI, and connectivity, smart hospitals have the potential to revolutionize the way healthcare is delivered, making it more personalized, proactive, and efficient than ever before.

### 2.2. AI-based solutions in smart hospitals and their implications for healthcare delivery

In the context of smart hospitals, integrating AI holds immense potential to revolutionize healthcare delivery and improve patient outcomes.^[8]^ AI-based solutions encompass various applications, including predictive analytics, personalized medicine, virtual assistants, and robotic surgery. By harnessing the power of machine-learning algorithms and advanced analytics, smart hospitals can extract valuable insights from vast amounts of data, leading to more informed decision-making and proactive healthcare interventions.^[9]^

One of the key benefits of AI-based solutions in smart hospitals is their ability to enhance diagnostic accuracy and efficiency. Machine-learning algorithms can analyze medical imaging data, such as MRI scans and X-rays, to identify patterns and anomalies indicative of disease or abnormalities.^[10]^ By automating the analysis process, AI algorithms can assist radiologists and other healthcare professionals in detecting diseases early, leading to timely interventions and improved patient outcomes. Furthermore, AI-based diagnostic tools can reduce the burden on healthcare providers and streamline workflow processes, enabling more efficient use of resources and reducing waiting times for patients.^[11]^

Another area where AI-based solutions are making a significant impact in smart hospitals is in the realm of personalized medicine. By analyzing patient data, including genetic information, medical history, and lifestyle factors, AI algorithms can tailor treatment plans to individual patients’ needs and preferences.^[12]^ This personalized approach to healthcare delivery improves treatment efficacy and enhances patient satisfaction and engagement. Additionally, AI-powered virtual assistants can provide patients personalized health recommendations, medication reminders, and lifestyle advice, empowering them to manage their health actively.^[13]^

Furthermore, AI-driven robotic surgery systems are revolutionizing the field of surgery by enabling precise and minimally invasive procedures. These systems use advanced robotics and AI algorithms to assist surgeons in performing complex surgical tasks with greater accuracy and precision.^[14]^ By reducing the risk of human error and improving surgical outcomes, AI-driven robotic surgery systems are transforming how surgeries are conducted in smart hospitals, leading to shorter recovery times, reduced complications, and improved patient satisfaction.^[15]^

In conclusion, AI-based solutions are poised to transform healthcare delivery in smart hospitals, offering opportunities to improve diagnostic accuracy, personalize treatment plans, and enhance surgical outcomes. By embracing AI technology, smart hospitals can optimize patient care delivery, improve operational efficiency, and ultimately enhance the overall quality of healthcare services. As AI continues to evolve and advance, its potential to revolutionize healthcare delivery in smart hospitals will only grow, ushering in a new era of precision medicine and patient-centered care.

### 2.3. The role of 5G in healthcare

5G technology represents a significant leap forward in wireless communications, offering unparalleled connectivity and data transfer speeds that have the potential to revolutionize healthcare delivery.^[1]^ One of the critical advantages of 5G is its ability to enhance connectivity by providing faster and more reliable network access, even in densely populated areas or remote locations.^[2]^ With 5G, healthcare providers can seamlessly transmit large volumes of data, such as medical images, patient records, and real-time monitoring data, without experiencing bottlenecks or disruptions.^[8]^

Moreover, 5G technology is characterized by its low latency, which refers to the delay between sending and receiving data packets over the network. This reduced latency ensures near-instantaneous communication between devices, enabling healthcare professionals to make timely decisions and respond to critical events in real-time. For example, milliseconds can make a significant difference in patient outcomes in emergencies or during surgical procedures, and 5G low-latency capabilities can help ensure that vital information reaches the right individuals without delay.^[9]^

In addition to low latency, 5G offers high bandwidth, allowing for the simultaneous transmission of large data streams faster than previous generations of wireless technology.^[5]^ This high-bandwidth enables bandwidth-intensive applications, such as high-definition video conferencing, remote surgery, and virtual reality-based training simulations, to seamlessly integrate into healthcare workflows. Healthcare providers can leverage these capabilities to conduct virtual consultations, collaborate with specialists across geographic boundaries, and deliver immersive training experiences to medical staff.^[10]^

The benefits of 5G for healthcare extend beyond enhanced connectivity and data transfer speeds. 5G technology also enables the proliferation of innovative use cases that potentially transform patient care and facility management.^[4]^ For instance, in patient care, 5G can facilitate remote monitoring and telemedicine services, allowing patients to receive virtual consultations, participate in remote rehabilitation programs, and access medical advice from the comfort of their homes. Similarly, 5G-powered IoT sensors and devices in facility management can monitor equipment performance, track inventory levels, and optimize energy consumption in real-time, leading to more efficient operations and cost savings.^[1]^

5G technology promises to revolutionize healthcare delivery by enabling faster, more reliable connectivity, low-latency communications, and high-bandwidth applications. As healthcare providers continue to explore the possibilities of 5G-enabled solutions, the potential to improve patient outcomes, enhance operational efficiency, and drive innovation in healthcare remains limitless.^[7]^

### 2.4. Innovations in patient care

5G technology is poised to revolutionize patient care by enabling remote monitoring and telemedicine services that transcend the limitations of traditional healthcare delivery models.^[5]^ With its high-speed connectivity and low-latency capabilities, 5G facilitates real-time data transmission and seamless communication between patients and healthcare providers, regardless of geographical distance.^[2]^

Remote patient monitoring (RPM) is one area where 5G holds immense potential to improve patient outcomes and enhance the quality of care. By leveraging wearable devices equipped with sensors, patients can continuously monitor vital signs, such as heart rate, blood pressure, and glucose levels, and transmit this data to healthcare professionals in real-time.^[4]^ With 5G high bandwidth and low latency, RPM systems can deliver accurate and timely information to healthcare providers, enabling early detection of health issues, proactive interventions, and personalized treatment plans tailored to individual patient needs.^[4]^

Telemedicine is another application of 5G technology that transforms how healthcare is delivered, particularly in remote or underserved areas where access to healthcare services is limited. With 5G-powered telemedicine platforms, patients can connect with healthcare providers via high-definition video conferencing, conduct virtual consultations, and receive medical advice without needing in-person visits.^[5]^ This improves patient care access and enhances convenience and flexibility, reducing the burden on healthcare facilities and minimizing appointment wait times.^[6]^

In addition to remote monitoring and telemedicine, 5G technology is driving the development of innovative healthcare solutions, such as wearable devices and augmented reality (AR) for surgical assistance. Wearable devices like smartwatches, fitness trackers, and biosensors have 5G connectivity, enabling continuous patient health metrics and biometric data monitoring.^[7]^ These wearable devices can alert users and healthcare providers to potential health issues in real-time, empowering individuals to take proactive steps to manage their health and well-being.^[3]^

AR is another groundbreaking application of 5G technology that revolutionizes surgical procedures and medical training. By overlaying digital information onto the physical world in real-time, AR technology provides surgeons with enhanced visualization and spatial awareness during complex procedures, improving precision and reducing the risk of errors.^[8]^ With 5G high-speed connectivity, AR-assisted surgery can be conducted remotely, allowing expert surgeons to guide and assist less experienced colleagues in different locations.^[1]^

Overall, 5G-enabled innovations in patient care are reshaping the healthcare landscape, enabling more personalized, accessible, and efficient healthcare delivery. By harnessing the power of 5G technology, healthcare providers can improve patient outcomes, enhance the patient experience, and ultimately transform how healthcare is delivered and experienced.

### 2.5. Improvements in facility management

Integrating 5G technology in facility management promises to revolutionize how healthcare facilities are monitored, managed, and maintained.^[9]^ With its high-speed connectivity and low latency, 5G facilitates real-time data analytics, enabling facility managers to access critical information instantaneously and make informed decisions to optimize operations.^[2]^

One essential application of 5G in facility management is real-time data analytics for monitoring and managing facility infrastructure. By deploying IoT sensors throughout the facility, 5G enables continuous data collection on various parameters such as temperature, humidity, air quality, and equipment performance.^[10]^ This data is then transmitted to a centralized platform where it is analyzed in real-time to identify anomalies, detect potential issues, and optimize resource allocation. For example, in a hospital setting, 5G-powered real-time data analytics can help identify areas of high energy consumption, monitor equipment utilization, and ensure compliance with regulatory requirements.^[3]^

In addition to real-time data analytics, 5G technology facilitates applications such as optimizing energy usage, asset tracking, and maintenance scheduling. With 5G-enabled energy management systems, facility managers can monitor energy consumption patterns, identify inefficiencies, and implement targeted interventions to reduce energy costs and minimize environmental impact.^[11]^ Similarly, 5G-powered asset-tracking solutions leverage IoT sensors and location-based services to track the movement and utilization of equipment, supplies, and personnel within the facility, improving inventory management and resource allocation.^[4]^ Moreover, 5G enables predictive maintenance scheduling by analyzing real-time equipment performance data, identifying potential issues before they escalate into costly failures, and optimizing maintenance schedules to minimize downtime and maximize operational efficiency.^[12]^

Adopting 5G-enabled facility management solutions offers significant cost savings and operational efficiencies for healthcare facilities. By leveraging real-time data analytics and predictive maintenance capabilities, facilities can reduce maintenance costs, extend equipment lifespan, and minimize unplanned downtime.^[13]^ Furthermore, optimizing energy usage through 5G-powered energy management systems can lead to substantial cost savings on utility bills and contribute to sustainability efforts by reducing carbon emissions. Overall, 5G-enabled facility management solutions empower healthcare facilities to operate more efficiently, reduce operational costs, and enhance the quality of patient care.^[5]^

### 2.6. Patient privacy and data security

In the era of smart hospitals and interconnected healthcare systems, protecting patient data is paramount to maintaining trust, ensuring confidentiality, and complying with regulatory requirements.^[2]^ As healthcare organizations increasingly rely on digital technologies and data-driven insights to deliver personalized care, safeguarding patient privacy and data security becomes even more critical.^[11]^

Protecting patient data in smart hospital environments cannot be overstated. Patient health information is susceptible and contains many personal and confidential details, including medical history, diagnoses, treatments, and genetic information. Unauthorized access to this data could lead to identity theft, medical fraud, and breaches of patient confidentiality, eroding trust between patients and healthcare providers.^[12]^

However, ensuring patient privacy and data security in a 5G-enabled healthcare ecosystem presents unique challenges. With the proliferation of connected devices and the rapid exchange of data over high-speed networks, the risk of data breaches and cyberattacks increases exponentially. Healthcare organizations must contend with threats such as malware, ransomware, and insider threats that can compromise the integrity and confidentiality of patient data.^[13]^

To address these challenges, healthcare organizations must implement robust cybersecurity measures and adopt best practices for data protection. This includes encrypting sensitive data both in transit and at rest, implementing access controls and authentication mechanisms to restrict unauthorized access, and regularly auditing and monitoring system activity to detect and mitigate security incidents promptly.^[14]^

Furthermore, healthcare organizations must ensure compliance with regulatory requirements governing the protection of patient data, such as the Health Insurance Portability and Accountability Act (HIPAA) in the United States.^[15]^ HIPAA sets stringent standards for safeguarding protected health information (PHI) and imposes penalties for noncompliance, including fines and legal consequences. Healthcare organizations must implement policies and procedures to ensure HIPAA compliance, including conducting risk assessments, implementing safeguards to protect PHI, and providing staff training on data security and privacy practices.^[15,16]^

### 2.7. Staff training and adoption

The successful integration of 5G-enabled technologies in patient care and facility management hinges on the preparedness and proficiency of healthcare staff.^[16]^ As healthcare organizations transition to 5G-enabled smart hospitals, there is a pressing need for comprehensive staff training and education to ensure that personnel are equipped with the knowledge and skills necessary to utilize these advanced technologies effectively.^[1]^

One of the primary challenges in implementing 5G-enabled technologies is overcoming staff resistance to technological changes. Healthcare professionals must be more responsive about adopting new technologies due to concerns about usability, workflow disruption, or perceived threats to job security.^[17]^ To address these concerns, healthcare organizations must invest in targeted training programs that teach staff to use 5G-enabled technologies and emphasize the benefits and potential impact on patient care and operational efficiency.^[17]^

Strategies for promoting staff adoption of 5G-enabled technologies include providing hands-on training sessions, offering online learning modules, and incorporating technology demonstrations into staff meetings and workshops.^[6]^ By engaging staff in the learning process and soliciting their feedback and input, healthcare organizations can foster a culture of innovation and continuous learning that encourages staff to embrace new technologies and adapt to changing work environments.^[18]^

Moreover, ongoing support and professional development play a crucial role in maximizing the benefits of 5G in smart hospitals. Healthcare organizations should establish dedicated support teams or help desks to assist staff with troubleshooting technical issues, answering questions, and providing guidance on best practices for using 5G-enabled technologies.^[8]^ Additionally, offering opportunities for continuing education, certification programs, and peer-to-peer knowledge sharing can help staff stay abreast of the latest developments in 5G technology and enhance their proficiency in using these tools effectively.^[19]^

By prioritizing staff training, adoption, and ongoing support, healthcare organizations can ensure that their workforce is well-equipped to leverage the full potential of 5G-enabled technologies in patient care and facility management.^[7]^ Through targeted training programs, strategic communication, and ongoing professional development initiatives, healthcare organizations can empower staff to embrace technological innovation and drive positive change in the delivery of healthcare services.^[20]^

### 2.8. Patient experience and satisfaction

Enhancing the patient experience is at the forefront of healthcare delivery, and 5G-enabled innovations play a pivotal role in achieving this goal.^[1]^ By leveraging advanced technologies such as telemedicine and remote monitoring, healthcare providers can significantly improve the convenience, accessibility, and quality of care for patients, ultimately leading to higher levels of satisfaction and improved health outcomes.^[21]^

Telemedicine, powered by 5G technology, offers patients unprecedented access to healthcare services, regardless of location or mobility constraints.^[6]^ Through high-definition video conferencing and real-time communication tools, patients can connect with healthcare providers for virtual consultations, follow-up appointments, and medical advice from the comfort of their homes.^[16]^ This eliminates the need for time-consuming and often costly travel to healthcare facilities and enhances access to care for individuals living in remote or underserved areas.^[22]^ Moreover, telemedicine empowers patients to take a more active role in managing their health by facilitating regular check-ins, medication management, and chronic disease management, leading to improved health outcomes and reduced hospitalizations.

Similarly, remote monitoring technologies enabled by 5G allow healthcare providers to track patient health metrics and vital signs remotely in real-time without needing in-person visits.^[15]^ Wearable devices, sensors, and mobile health apps can continuously monitor parameters such as heart rate, blood pressure, glucose levels, and activity levels, alerting healthcare providers to potential health issues or changes in condition.^[2]^ This proactive approach to monitoring enhances patient safety and early intervention and improves patient engagement and adherence to treatment plans, leading to better health outcomes and reduced healthcare costs.

Central to improving the patient experience in smart hospitals is integrating patient feedback and satisfaction surveys into healthcare delivery processes.^[6]^ By soliciting patient feedback about their experiences, preferences, and concerns, healthcare providers can gain valuable insights into areas for improvement and opportunities to enhance the quality of care.^[9]^ Patient feedback can inform decisions about service delivery, facility design, communication protocols, and clinical practices, ultimately leading to more patient-centered and personalized care experiences.^[23]^ Moreover, by regularly assessing patient satisfaction through surveys and feedback mechanisms, healthcare providers can benchmark performance, track progress over time, and identify trends or patterns that require attention.

### 2.9. Collaboration and partnerships

Collaboration between healthcare providers, technology vendors, and other stakeholders is essential for successfully implementing 5G-enabled solutions in healthcare.^[6]^ The complexity and interdisciplinary nature of healthcare technology require expertise from various domains, including healthcare delivery, telecommunications, information technology, and regulatory compliance. By fostering partnerships and collaboration, stakeholders can leverage their strengths and resources to drive innovation, overcome challenges, and accelerate the adoption of 5G in healthcare.^[24]^

Successful partnerships between healthcare providers and technology vendors have led to innovative applications of 5G in healthcare. For example, collaborations between hospitals and telecommunications companies have resulted in the development of 5G-powered telemedicine platforms, remote monitoring systems, and virtual care solutions.^[4]^ These partnerships leverage the expertise of healthcare providers in clinical care delivery with the technical capabilities of technology vendors to create seamless and integrated healthcare experiences for patients and providers alike.^[5]^

Moreover, cross-industry collaborations can potentially drive further advancements in smart hospital technologies. Innovative solutions that address complex healthcare challenges and improve patient outcomes can be developed by bringing together stakeholders from diverse industries, such as telecommunications, biotechnology, pharmaceuticals, and consumer electronics.^[7]^ For example, collaborations between healthcare providers and consumer electronics companies have led to the development of wearable health devices with integrated 5G connectivity, allowing for real-time health monitoring and data transmission.^[6]^

Additionally, partnerships between healthcare providers and academic institutions can facilitate research and development efforts to explore the potential of 5G in healthcare.^[6]^ By combining the expertise of researchers, clinicians, and industry partners, these collaborations can drive innovation, validate new technologies, and translate research findings into practical applications that benefit patients and healthcare providers.^[8]^

### 2.10. Regulatory landscape

Implementing 5G technology in healthcare settings is subject to regulatory frameworks that govern the use and deployment of wireless communications technologies and patient data and privacy protection.^[3]^ While 5G offers immense potential to transform healthcare delivery, regulatory requirements are crucial in shaping the adoption and deployment of 5G-enabled solutions in smart hospitals.^[1]^

One critical regulatory framework governing the implementation of 5G in healthcare is the Federal Communications Commission (FCC) regulations in the United States. The FCC oversees the allocation and licensing of spectrum for wireless communications, including 5G networks.^[6]^ Healthcare organizations must ensure compliance with FCC regulations when deploying 5G-enabled devices and infrastructure to avoid interference with other wireless systems and ensure the reliability and security of communication networks.^[2]^

In addition to FCC regulations, healthcare providers must comply with data protection and privacy regulations, such as the HIPAA in the United States.^[8]^ HIPAA sets stringent standards for safeguarding PHI and imposes penalties for noncompliance, including fines and legal consequences. Healthcare organizations must implement policies and procedures to ensure HIPAA compliance when collecting, storing, and transmitting patient data over 5G networks to protect patient privacy and confidentiality.^[3]^

Furthermore, regulatory requirements influence the adoption and deployment of 5G-enabled solutions in smart hospitals by imposing certification and interoperability standards and requirements for cybersecurity and data encryption.^[15]^ Healthcare organizations must ensure that 5G-enabled devices and applications meet regulatory standards for safety, security, and interoperability to minimize risks and ensure the reliability and effectiveness of healthcare delivery.^[4]^

Recent policy developments have also shaped the regulatory landscape for 5G in healthcare, with governments worldwide prioritizing investments in digital infrastructure and healthcare technology to improve access to care and enhance patient outcomes.^[12]^ For example, the European Union Digital Health Strategy aims to promote digital technologies, including 5G, to support innovation in healthcare delivery and improve cross-border health data interoperability.^[13]^ Similarly, initiatives such as the National Institutes of Health All of Us Research Program in the United States are leveraging 5G technology to enable data sharing and collaboration among researchers, healthcare providers, and patients to advance precision medicine and personalized healthcare.^[5]^

### 2.11. Global perspectives

The adoption of 5G-enabled smart hospital technologies varies significantly across regions worldwide, reflecting variations in healthcare infrastructure, regulatory environments, and technological readiness.^[14]^

In North America, particularly in the United States and Canada, healthcare organizations have been at the forefront of adopting 5G-enabled technologies to enhance patient care and operational efficiency.^[15]^ With robust healthcare infrastructure and significant investments in digital health initiatives, North American healthcare systems have embraced innovations such as telemedicine, remote monitoring, and predictive analytics powered by 5G technology.^[16]^ Regulatory frameworks like HIPAA in the United States govern using 5G in healthcare to ensure patient privacy and data security.^[20]^ Noteworthy initiatives include the Mayo Clinic Center for Digital Health, which leverages 5G technology to deliver remote consultations, virtual care, and medical education initiatives to patients and healthcare providers across the region.^[17]^

In Europe, countries like the United Kingdom, Germany, and Sweden are exploring the potential of 5G-enabled smart hospital technologies to improve healthcare delivery and patient outcomes.^[18]^ While healthcare infrastructure varies across European countries, initiatives such as the European Commission Digital Health and Care Strategy aim to promote the adoption of digital technologies, including 5G, to support innovation in healthcare and enhance cross-border interoperability of health data.^[21]^ Regulatory frameworks, such as the General Data Protection Regulation, govern using 5G in healthcare to ensure data protection and privacy. Best practices from European healthcare systems include deploying 5G-powered telemedicine platforms, remote monitoring systems, and digital health records to improve access to care and empower patients to manage their health proactively.^[19]^

In Asia-Pacific, countries such as China, South Korea, and Japan are leading the way in adopting 5G-enabled smart hospital technologies to address the growing demand for healthcare services and improve health outcomes.^[20]^ With rapidly expanding healthcare infrastructure and investments in digital health initiatives, Asian healthcare systems are leveraging 5G technology to deliver innovative solutions such as AI-powered diagnostics, robotic surgery, and RPM.^[4]^ Regulatory environments vary across Asian countries, with initiatives such as China National Health Commission Digital Health Strategy promoting the integration of 5G and AI technologies into healthcare delivery.^[21]^ Noteworthy initiatives include deploying 5G-enabled medical robots in hospitals to assist with patient care, disinfection, and logistics, using 5G-powered drones to deliver medical supplies, and conducting emergency response operations in remote areas.^[6]^

In summary, the adoption of 5G-enabled smart hospital technologies varies across regions worldwide, reflecting regional variations in healthcare infrastructure, regulatory environments, and technological readiness. While healthcare systems face unique challenges and opportunities, international collaboration and knowledge sharing can help accelerate the adoption of 5G technology and drive continuous innovation in healthcare delivery globally.^[7]^

### 2.12. Research and innovation

Ongoing research efforts and innovative projects are continuously pushing the boundaries of 5G technology in healthcare, focusing on advancing patient care, improving operational efficiency, and driving medical breakthroughs.^[8]^

In remote surgery, researchers are exploring the potential of 5G technology to enable real-time, high-definition video streaming and ultra-low-latency communication between surgeons and robotic surgical systems.^[5]^ Recent breakthroughs in this area include successful demonstrations of remote surgical procedures conducted over 5G networks, showcasing the potential for surgeons to perform complex operations from remote locations with unparalleled precision and accuracy. These advancements can expand access to specialized surgical care for patients in underserved areas and improve outcomes for complex surgical procedures.^[6]^

Predictive analytics powered by 5G technology are revolutionizing healthcare by enabling early detection of diseases, personalized treatment planning, and proactive interventions to prevent adverse health events.^[7]^ Researchers are leveraging the massive amounts of data generated by 5G-enabled devices and sensors to develop advanced machine-learning algorithms and predictive models to analyze real-time health data and identify patterns and trends indicative of potential health risks.^[9]^ Recent breakthroughs in predictive analytics have led to predictive tools for identifying patients at risk of developing chronic conditions, predicting hospital readmissions, and optimizing treatment regimens based on individual patient characteristics and preferences.^[10]^

Furthermore, emerging technologies such as edge computing and blockchain hold tremendous promise for enhancing the capabilities of 5G-enabled smart hospitals. Edge computing enables data processing and analysis closer to the point of data generation, reducing latency and enabling real-time decision-making in healthcare settings.^[11]^ By leveraging edge computing infrastructure, healthcare providers can deploy AI algorithms to analyze medical imaging data, monitor patient vitals, and detect anomalies in real-time, leading to faster diagnoses and more timely interventions.^[4]^ Additionally, blockchain technology offers a secure and decentralized platform for storing, sharing, and managing healthcare data, ensuring data integrity, privacy, and interoperability across disparate systems. By integrating blockchain with 5G networks, healthcare organizations can create transparent and auditable records of patient data transactions, streamline data exchange between providers and patients, and enhance trust and accountability in healthcare delivery.^[8]^

### 2.13. Scalability and interoperability

Scaling up 5G-enabled smart hospital solutions across multiple facilities and healthcare systems presents challenges and opportunities.^[5]^ While the deployment of 5G technology offers immense potential to improve patient care and operational efficiency, ensuring seamless integration and interoperability across diverse healthcare environments is essential for realizing the full benefits of these solutions.^[10]^

One of the primary challenges related to scalability is the complexity of implementing 5G-enabled smart hospital solutions across multiple facilities with varying infrastructure, resources, and organizational structures.^[14]^ Healthcare organizations must consider network coverage, bandwidth requirements, and device compatibility when scaling up 5G deployments to ensure consistent performance and reliability across different locations.^[10]^ Additionally, cost considerations, regulatory requirements, and data privacy concerns may further complicate scaling up 5G-enabled solutions, particularly for large healthcare systems with numerous facilities and stakeholders.^[11]^

However, scalability also presents opportunities for healthcare organizations to leverage economies of scale, standardize processes, and streamline operations through centralized management and coordination of 5G-enabled technologies.^[17]^ By investing in scalable infrastructure and modular solutions that can be easily deployed and integrated across multiple facilities, healthcare organizations can maximize efficiency, reduce costs, and improve the quality of care for patients across the continuum of care.^[12]^

Interoperability standards are critical in ensuring seamless integration of different technologies and devices within smart hospital environments. With the proliferation of connected devices, sensors, and systems, interoperability becomes essential for enabling data exchange, communication, and collaboration among disparate healthcare IT systems and platforms.^[17]^ Interoperability standards such as HL7, FHIR, and DICOM define common data formats, protocols, and interfaces that facilitate the exchange of health information and promote interoperability across different healthcare systems and vendors.^[13]^

Promoting interoperability and compatibility among diverse healthcare IT systems and platforms requires strategic planning, collaboration, and investment in interoperability infrastructure and technologies.^[16]^ Healthcare organizations can adopt strategies such as adopting open standards and APIs, implementing data normalization and aggregation tools, and participating in interoperability initiatives and consortiums to promote data sharing and exchange. Additionally, interoperability testing, certification programs, and vendor collaborations can ensure compatibility and seamless integration of 5G-enabled technologies within smart hospital environments.^[14]^

### 2.14. Community engagement and public outreach

Community engagement and public outreach are crucial in promoting awareness and acceptance of 5G-enabled smart hospitals. By involving patients, caregivers, and the general public in discussions about the benefits of 5G technology in healthcare, healthcare organizations can build trust, address concerns, and foster support for adopting innovative technologies.^[15]^

Initiatives aimed at educating patients, caregivers, and the general public about the benefits of 5G technology in healthcare include informational campaigns, public forums, and educational materials designed to explain the potential applications of 5G in improving patient care and enhancing healthcare delivery.^[13]^ These initiatives often highlight the advantages of 5G-enabled telemedicine, remote monitoring, and personalized medicine in enabling more accessible, convenient, and efficient healthcare services.^[16]^ By providing clear and concise information about how 5G technology works and its potential impact on healthcare, healthcare organizations can empower patients and caregivers to make informed decisions about their health and engage more actively in their care.

Moreover, involving local communities in designing and implementing smart hospital solutions can help healthcare organizations better understand the unique needs, preferences, and cultural sensitivities of the populations they serve.^[6]^ Community engagement strategies include conducting focus groups, surveys, and town hall meetings to solicit feedback and input from community members on how 5G-enabled technologies can be tailored to meet their specific needs and preferences. By incorporating community input into the design and implementation of smart hospital solutions, healthcare organizations can ensure that these technologies are accessible, inclusive, and responsive to the needs of diverse populations.^[17]^

Opportunities for involving local communities in designing and implementing smart hospital solutions include partnering with community organizations, local governments, and grassroots advocacy groups to co-create initiatives that address community health priorities and improve access to care.^[8]^ For example, healthcare organizations may collaborate with community health centers, faith-based organizations, and schools to deploy mobile health clinics equipped with 5G-enabled telemedicine capabilities to underserved areas, providing residents access to primary care services and specialty consultations without traveling or long wait times.^[1]^ By engaging with local stakeholders and leveraging community resources, healthcare organizations can create sustainable and impactful solutions that address the healthcare needs of their communities.^[18]^

### 2.15. Sustainability and environmental impact

5G-enabled smart hospitals have the potential to significantly contribute to sustainability efforts through energy-efficient operations and reduced resource consumption. By leveraging advanced technologies and innovative solutions, smart hospitals can optimize energy usage, minimize waste, and mitigate environmental impact while delivering high-quality healthcare services.^[19]^

Telemedicine and remote monitoring, enabled by 5G technology, offer environmental benefits by reducing the need for patient travel and hospital visits. By providing virtual consultations and remote monitoring capabilities, healthcare providers can deliver care to patients in their homes, reducing the carbon footprint associated with transportation and hospital visits.^[6]^ This reduces greenhouse gas emissions and air pollution and conserves valuable resources such as fuel and healthcare infrastructure. Additionally, telemedicine and remote monitoring empower patients to take a more active role in managing their health, leading to better health outcomes and reduced healthcare utilization.^[20]^

Initiatives aimed at minimizing the carbon footprint of healthcare facilities through adopting renewable energy sources and green building practices are becoming increasingly prevalent in the healthcare industry.^[5]^ Smart hospitals can incorporate renewable energy technologies such as solar panels, wind turbines, and geothermal systems to generate clean and sustainable energy onsite, reducing reliance on fossil fuels and conventional energy sources. Additionally, green building practices such as energy-efficient design, passive heating and cooling, and sustainable materials can reduce energy consumption, improve indoor air quality, and enhance overall environmental sustainability.^[21]^

Furthermore, smart hospitals can implement smart energy management systems and IoT-enabled devices to monitor and optimize energy usage in real-time, identifying efficiency improvements and cost savings opportunities.^[10]^ By leveraging data analytics and machine-learning algorithms, healthcare facilities can optimize HVAC systems, lighting, and equipment usage to minimize energy waste and reduce operational costs. Smart waste management systems can also help healthcare facilities track and manage waste streams more efficiently, reducing landfill waste and promoting recycling and composting practices.^[22]^

### 2.16. Disaster preparedness and emergency response

5G-enabled smart hospitals are critical in enhancing disaster preparedness and response efforts by leveraging advanced technologies to improve situational awareness, coordination, and communication during emergencies.^[15]^ Through real-time data analytics, remote monitoring capabilities, and innovative solutions, smart hospitals can effectively respond to natural disasters, pandemics, and other crises, ensuring the safety and well-being of patients, staff, and communities.^[23]^

Real-time data analytics and remote monitoring capabilities enable smart hospitals to gather, analyze, and share vital information during emergencies, providing healthcare providers and emergency responders with actionable insights and situational awareness.^[5]^ By integrating data from various sources, such as patient records, environmental sensors, and public health surveillance systems, smart hospitals can identify patterns, detect anomalies, and anticipate emerging threats, enabling proactive decision-making and resource allocation.^[8]^ This real-time data monitoring and analysis allow healthcare organizations to respond swiftly to emergencies, coordinate resources effectively, and mitigate risks to patient safety and public health.^[23]^

Case studies of smart hospitals leveraging 5G technology to respond to disasters demonstrate advanced technologies’ transformative impact on emergency preparedness and response.^[5]^ For example, during the Coronavirus Disease 2019 pandemic, smart hospitals utilized 5G-enabled telemedicine platforms and remote monitoring systems to provide virtual consultations, monitor patient vitals remotely, and manage surge capacity, reducing virus transmission risk and strain on healthcare resources.^[24]^ Additionally, smart hospitals deployed drones with 5G connectivity to deliver medical supplies, conduct aerial surveys, and provide situational awareness in hard-to-reach or disaster-affected areas, enabling rapid response and disaster relief efforts.

Furthermore, smart hospitals have leveraged 5G technology to enhance communication and collaboration among healthcare providers, emergency responders, and public health agencies during emergencies.^[12]^ By enabling high-speed, low-latency communication networks, 5G facilitates real-time video conferencing, data sharing, and decision support tools, enabling seamless coordination and information exchange among stakeholders. This enhanced communication and collaboration allow healthcare organizations to mobilize resources, coordinate care delivery, and respond effectively to emergencies, improving outcomes for patients and communities.^[25]^

## 3. Case studies

### 3.1. Case Study 1: 5G-powered telemedicine for RPM

In a bustling metropolitan area, a smart hospital embraced the transformative potential of 5G technology to revolutionize RPM and telemedicine. Facing the challenge of providing comprehensive care to patients across vast geographical regions, the hospital sought innovative solutions to bridge the distance between patients and healthcare providers.

Utilizing 5G-enabled telemedicine platforms and IoT devices, the hospital implemented an RPM system that allowed healthcare professionals to monitor patients’ vital signs, medication adherence, and overall health status in real-time. Patients with wearable devices, such as smartwatches and biosensors, could securely transmit their health data to the hospital centralized monitoring system via ultra-fast 5G networks.

One notable case involved a patient living in a rural area, miles from the nearest healthcare facility. Suffering from a chronic heart condition, the patient required continuous monitoring and timely intervention to manage their condition effectively. With the implementation of 5G-powered telemedicine, the patient could receive personalized care from the comfort of their home.

The patient health status was closely monitored through daily remote monitoring of vital signs and regular video consultations with cardiologists, and treatment adjustments were made promptly as needed. In the event of abnormalities or emergencies, healthcare providers could intervene quickly, thanks to the low-latency connectivity provided by 5G networks, ensuring timely and life-saving interventions.

Furthermore, the hospital utilized AR technology powered by 5G to facilitate virtual consultations and surgical assistance. Surgeons could remotely guide and assist colleagues in complex surgical procedures, leveraging high-definition video streams and interactive AR overlays to visualize patient anatomy and surgical instruments in real-time.

The implementation of 5G-powered telemedicine improved patient outcomes and enhanced operational efficiency and resource utilization at the hospital. By reducing the need for in-person consultations and hospital visits, the hospital could optimize bed occupancy rates, minimize wait times, and allocate resources more effectively to needy patients.

In conclusion, this case study exemplifies the transformative impact of 5G technology on smart hospitals, particularly in RPM and telemedicine. By leveraging ultra-fast connectivity, real-time data transmission, and advanced technologies such as AR, smart hospitals can overcome geographical barriers, expand access to healthcare services, and deliver personalized care to patients wherever they may be. As 5G technology continues to evolve, the possibilities for innovation in healthcare delivery are boundless, promising a future where distance is no longer a barrier to quality care.

### 3.2. Case Study 2: Enhancing surgical precision with 5G-enabled robotic surgery

In a state-of-the-art smart hospital, integrating 5G technology has transformed the landscape of surgical care, particularly in robotic surgery. To enhance surgical precision and patient outcomes, the hospital embarked on a journey to leverage the capabilities of 5G-enabled robotic surgery systems.

One notable case involved a patient diagnosed with a complex cardiac condition requiring intricate surgical intervention. Traditionally, such procedures posed significant challenges due to the need for precise maneuvers and real-time feedback during surgery. However, with the adoption of 5G-enabled robotic surgery, the hospital surgical team was equipped with advanced tools and technologies to navigate these challenges confidently.

Utilizing robotic surgery systems powered by ultra-fast 5G connectivity, the surgical team could perform minimally invasive procedures with unparalleled precision and control. The high-definition video streams and real-time data transmission facilitated by 5G networks allowed surgeons to visualize patient anatomy in exquisite detail and make precise adjustments during the procedure.

During the surgery, the robotic system AI-driven algorithms analyzed the patient vital signs and surgical parameters in real-time, providing the surgical team with valuable insights and predictive analytics to optimize surgical outcomes. With AI assistance, surgeons could anticipate potential complications and adjust their approach accordingly, ensuring the best possible outcome for the patient.

One of the critical advantages of 5G-enabled robotic surgery was its ability to overcome geographical barriers and facilitate remote surgical assistance. In this case, renowned cardiac surgeons worldwide collaborated virtually. They guided the local surgical team in real-time, leveraging the high-speed connectivity and low-latency communication enabled by 5G networks.

The successful completion of the surgery demonstrated the transformative impact of 5G-enabled robotic surgery on patient care and surgical outcomes. By harnessing the power of 5G technology, the hospital achieved unprecedented levels of surgical precision, reduced the risk of complications, and improved patient recovery times.

Furthermore, adopting 5G-enabled robotic surgery systems resulted in tangible benefits for the hospital, including enhanced operational efficiency, reduced surgical times, and optimized resource utilization. As a result, the hospital increased patient throughput, minimized wait times, and improved overall patient satisfaction.

In conclusion, this case study illustrates the profound impact of 5G technology on surgical care in smart hospitals. By leveraging ultra-fast connectivity, real-time data transmission, and AI-driven algorithms, smart hospitals can revolutionize surgical procedures, enhance patient outcomes, and pave the way for a new era of precision medicine. As 5G technology continues to evolve, the possibilities for innovation in surgical care are limitless, promising a future where every patient receives the highest quality of care, no matter where they are located.

### 3.3. Case Study 3: Revolutionizing emergency response with 5G-powered disaster management

In a bustling urban setting prone to natural disasters, a forward-thinking smart hospital implemented innovative 5G-enabled solutions to revolutionize disaster management and emergency response. The hospital proactive approach to leveraging technology in emergencies was tested during a recent natural disaster—a devastating earthquake that struck the region.

Utilizing 5G-powered real-time data analytics and IoT sensors, the hospital disaster management team was able to rapidly assess the impact of the earthquake and prioritize response efforts. Within minutes of the disaster, data streams from seismic sensors, building monitoring systems, and patient tracking devices were aggregated and analyzed in real-time, providing crucial insights into the extent of damage and the location of affected areas.

One critical aspect of the hospital emergency response strategy involved deploying 5G-enabled telemedicine units to provide remote medical assistance to injured individuals in hard-to-reach areas. With high-definition video cameras, biometric sensors, and telepresence capabilities, these units enabled healthcare professionals to assess and triage patients onsite, facilitating timely interventions and evacuations.

In one particularly challenging scenario, a remote village in a mountainous region was cut off from traditional medical facilities due to landslide damage caused by the earthquake. Using 5G-powered telemedicine units, the hospital emergency response team established a virtual connection with the village makeshift medical clinic, providing real-time guidance and support to local healthcare providers.

Additionally, the hospital leveraged AR technology powered by 5G to assist search and rescue teams to locate and extract survivors trapped in collapsed buildings. AR overlays projected onto rescue workers’ smart glasses provided vital information about structural integrity, potential hazards, and the location of survivors, enabling more efficient and coordinated rescue efforts.

The successful deployment of 5G-enabled disaster management solutions resulted in significant improvements in emergency response capabilities and patient outcomes. By leveraging real-time data analytics, telemedicine, and AR technology, the hospital could expedite rescue operations, provide timely medical assistance, and minimize the loss of life and injury.

Furthermore, implementing 5G-powered disaster management solutions showcased the hospital commitment to innovation and preparedness in adversity. By embracing cutting-edge technologies, smart hospitals can enhance their resilience to natural disasters, improve community safety, and ensure continuity of care in times of crisis.

In conclusion, this case study highlights the transformative impact of 5G technology on emergency response and disaster management in smart hospitals. By leveraging ultra-fast connectivity, real-time data analytics, and advanced technologies such as telemedicine and AR, smart hospitals can strengthen their capacity to respond effectively to emergencies, save lives, and mitigate the impact of natural disasters. As 5G technology continues to evolve, the potential for innovation in disaster management is limitless, promising a safer and more resilient future for communities worldwide.

### 3.4. Continuous improvement and innovation

Fostering a continuous improvement and innovation culture is paramount to driving excellence in patient care and facility management in smart hospital environments.^[20]^ By embracing a mindset of experimentation, learning, and knowledge sharing, healthcare providers and technology partners can identify opportunities for improvement, implement innovative solutions, and adapt to changing healthcare needs and technological advancements.^[26–29]^

Strategies for promoting experimentation, learning, and knowledge sharing among healthcare providers and technology partners include creating interdisciplinary teams, fostering collaboration and communication, and providing professional development and training opportunities.^[30,31]^ By bringing together individuals with diverse backgrounds, expertise, and perspectives, healthcare organizations can harness their workforce collective intelligence and creativity to generate new ideas, solve complex problems, and drive innovation.^[32]^ Additionally, establishing platforms for sharing best practices, lessons learned, and success stories enables healthcare providers and technology partners to learn from each other experiences, replicate successful strategies, and avoid common pitfalls.^[28]^

Feedback mechanisms, performance metrics, and quality improvement initiatives drive ongoing innovation and excellence in patient care and facility management.^[33]^ By soliciting feedback from patients, caregivers, and staff, healthcare organizations can identify areas for improvement, address concerns, and prioritize initiatives that have the most significant impact on patient outcomes and operational efficiency.^[34–36]^ Performance metrics such as patient satisfaction scores, readmission rates, and operational key performance indicators provide objective performance measures and enable healthcare organizations to track progress, benchmark against industry standards, and identify areas for improvement.^[37,38]^ Quality improvement initiatives such as lean manufacturing and Six Sigma methodologies, Total Quality Management, and Continuous Process Improvement empower healthcare providers and technology partners to systematically identify inefficiencies, streamline workflows, and optimize resource utilization, driving continuous improvement and innovation in smart hospital environments.^[39,40]^

## 4. Conclusion and call to action

The advent of 5G-enabled smart hospitals heralds a new healthcare delivery and facility management era, empowered by cutting-edge technologies and innovative solutions. This article explored how 5G technology transforms patient care, disaster preparedness, environmental sustainability, and continuous improvement in healthcare settings.

Key insights gleaned include the pivotal role of 5G in enhancing connectivity, enabling real-time data analytics, and facilitating remote monitoring and telemedicine. By leveraging these capabilities, smart hospitals can improve patient outcomes, enhance operational efficiency, and drive innovation in healthcare delivery.

Stakeholders across the healthcare ecosystem must embrace 5G technology as a catalyst for transformational change. By collaborating, innovating, and investing in 5G-enabled smart hospital solutions, we can collectively advance the future of healthcare and improve the lives of patients and communities.

Therefore, I encourage healthcare providers, technology vendors, policymakers, and investors to join forces in harnessing the power of 5G to revolutionize healthcare delivery and facility management. Together, let us embrace innovation, drive excellence, and create a brighter future where smart hospitals serve as beacons of hope, healing, and resilience in the face of adversity.

## Acknowledgments

We want to express our gratitude to all the individuals and organizations who have contributed to the creation of this article. Special thanks to the healthcare providers, researchers, and technology experts whose insights and expertise have informed the content of this publication. We also acknowledge the patients and communities whose experiences and feedback have enriched our understanding of the role of 5G technology in healthcare delivery. Additionally, we extend our appreciation to the reviewers and editors who have provided valuable feedback and guidance throughout the development of this article. Finally, we thank our readers for their interest in this topic and commitment to advancing healthcare innovation.

## Author contributions

**Conceptualization:** Chukwuka Elendu, Tochi C. Elendu, Ijeoma D. Elendu.

**Data curation:** Chukwuka Elendu, Tochi C. Elendu, Ijeoma D. Elendu.

**Formal analysis:** Chukwuka Elendu, Tochi C. Elendu, Ijeoma D. Elendu.

**Funding acquisition:** Chukwuka Elendu, Tochi C. Elendu, Ijeoma D. Elendu.

**Investigation:** Chukwuka Elendu, Tochi C. Elendu, Ijeoma D. Elendu.

**Methodology:** Chukwuka Elendu, Tochi C. Elendu, Ijeoma D. Elendu.

**Project administration:** Chukwuka Elendu, Tochi C. Elendu, Ijeoma D. Elendu.

**Resources:** Chukwuka Elendu, Tochi C. Elendu, Ijeoma D. Elendu.

**Software:** Chukwuka Elendu, Tochi C. Elendu, Ijeoma D. Elendu.

**Supervision:** Chukwuka Elendu, Tochi C. Elendu, Ijeoma D. Elendu.

**Validation:** Chukwuka Elendu, Tochi C. Elendu, Ijeoma D. Elendu.

**Visualization:** Chukwuka Elendu, Tochi C. Elendu, Ijeoma D. Elendu.

**Writing – original draft:** Chukwuka Elendu, Tochi C. Elendu, Ijeoma D. Elendu.

**Writing – review & editing:** Chukwuka Elendu, Tochi C. Elendu, Ijeoma D. Elendu.
